# The flap endonuclease-1 promotes cellular tolerance to a chain-terminating nucleoside analog, alovudine, by counteracting the toxic effect of 53BP1

**DOI:** 10.1093/nar/gkaf617

**Published:** 2025-07-18

**Authors:** Md Bayejid Hosen, Ryotaro Kawasumi, Kouji Hirota

**Affiliations:** Department of Chemistry, Graduate School of Science, Tokyo Metropolitan University, Minamiosawa 1-1, Hachioji-shi, Tokyo 192-0397, Japan; Department of Chemistry, Graduate School of Science, Tokyo Metropolitan University, Minamiosawa 1-1, Hachioji-shi, Tokyo 192-0397, Japan; Department of Chemistry, Graduate School of Science, Tokyo Metropolitan University, Minamiosawa 1-1, Hachioji-shi, Tokyo 192-0397, Japan

## Abstract

Chain-terminating nucleoside analogs (CTNAs) are frequently used as antiviral and anticancer agents. CTNAs are incorporated into the end of nascent DNA during DNA synthesis and inhibit subsequent DNA polymerization, thereby restricting the proliferation of viruses and cancer cells. Alovudine, a thymidine analog, exerts chain-termination effects and is used as an antiviral drug. However, the mechanisms underlying cellular tolerance to alovudine have not been fully elucidated. Here, we show that flap endonuclease-1 (Fen1) and p53 binding protein-1 (53BP1) counteractively mitigate the chain-terminating effect of alovudine. We found that the cells deficient in Fen1 (*FEN1*^−/−^) showed stronger chain-terminating effects of alovudine with augmented DNA damage than wild-type cells, leading to their alovudine hypersensitivity. Moreover, we found that the Okazaki fragment maturation was critically slowed in *FEN1*^−/−^ cells when cells were challenged with alovudine. Remarkably, the loss of 53BP1 rescued these phenotypes. We found that 53BP1 formed sub-nuclear foci upon alovudine treatment and these 53BP1 foci were critically increased in *FEN1*^−/−^ cells, indicating that Fen1 suppresses alovudine-mediated toxic 53BP1 subnuclear foci thereby promoting Okazaki fragment maturation and suppressing DNA damage. In this study, we uncovered the previously unappreciated role of Fen1 in the suppression of toxic action of 53BP1 at alovudine-incorporated Okazaki fragment.

## Graphical Abstract

**Graphical Abstract:**
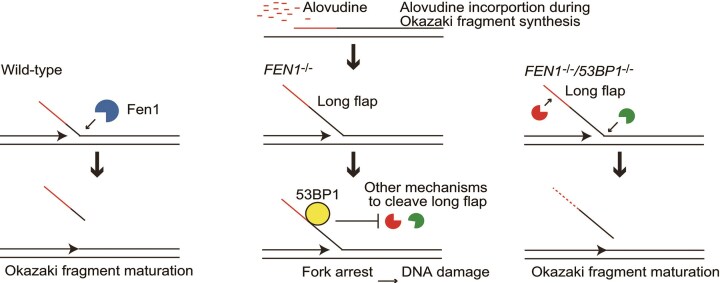

## Introduction

Chain-terminating nucleoside analogs (CTNAs) have been frequently used as drugs for the treatment of virus infection and cancers [1]. CTNAs incorporated into DNA interfere with DNA replication of viruses and cancer cells. CTNAs halt replication via following two distinct mechanisms [2]. First, in the chain-termination of replication, CTNAs incorporated into the end of nascent DNA inhibit subsequent polymerization reactions. Second, the subsequent round of DNA replication is stalled on the CTNA-incorporated template. Since viral DNA/RNA polymerases are more prone to incorporate CTNAs into their genomes than replicative DNA polymerases of host cells, the CTNA medications can restrict viral genome synthesis and ultimately their proliferations [3, 4]. Among CTNAs, alovudine efficiently inhibits viral reverse transcriptase and was initially tested as an anti-viral drug in the 1990s [5]. This drug effectively restricts the proliferation of human immunodeficiency virus (HIV) and is thus used frequently for the treatment of HIV [5, 6]. We previously revealed that alovudine can be incorporated into the end of the nascent strand thereby stalling the replication fork progression, with BRCA1 and flap endonuclease-1 (Fen1) playing a dominant role in cellular resistance to alovudine [7]. We demonstrated the critical role of BRCA1 in the removal of alovudine from the nascent strand [7]. However, the molecular mechanisms of how Fen1 contributes to the cellular tolerance to alovudine have not been elucidated.

Our primary study on alovudine identified the critical role of BRCA1 in suppressing DNA damage upon alovudine treatment and ultimately in the cellular tolerance to alovudine [7]. We found that BRCA1 suppresses DNA damage in the following two ways: First, BRCA1 suppresses chain-terminating effects by eliminating alovudine from the end of nascent DNA. Second, BRCA1 promotes homologous recombination (HR) to restart stalled replication on the alovudine-incorporated template [7]. We also found that the loss of p53 binding protein-1 (53BP1), a central mediator of DNA double-strand breaks (DSBs) signaling [8] in *BRCA1*^−/−^ cells restored their alovudine-hypersensitivity to nearly wild-type level as a result of the restoration of the functionality of HR [9], suggesting that the BRCA1’s second role (promoting HR) is critical for the cellular tolerance to alovudine.

Fen1 is an endonuclease that preferentially cleaves 5′-flapped structured DNA [10–12]. Fen1 plays a role in Okazaki fragment maturation during lagging strand synthesis by processing RNA primers dissociated through the subsequent Okazaki fragment synthesis by polymerase δ [13]. The Okazaki fragment maturation is mediated by two distinct pathways: the short flap pathway mediated by Fen1, and the long flap pathway mediated by Dna2 [14, 15]. 53BP1 is involved in the latter process, as 53BP1 interacts with RNA–DNA chimeric structure in the Okazaki fragment through its RNA binding domain, and then mediates Dna2-dependent Okazaki fragment processing [16]. The loss of Fen1 led to an increase of 53BP1 at Okazaki fragment as a result of augmentation of unligated Okazaki fragment, suggesting their compensatory action in the Okazaki fragment maturation [16]. In addition to its role in the Okazaki fragment maturation, Fen1 also plays a role in long patch base excision repair (BER), in which it contributes to eliminating 5′ flap structures created during gap-filling DNA synthesis [17, 18]. Fen1 is thus inevitable to sustain genomic stability and integrity, and the association of Fen1 mutation with various human cancers has also been reported [19–21].

In this study, we investigated the role of Fen1 in mitigating the chain-terminating effect of alovudine. We found that *FEN1*^−/−^ cells exhibited pronouncedly reduced replication fork progression kinetics with augmented DNA damage upon alovudine challenge compared to wild-type cells. Moreover, we found that Okazaki fragment maturation was critically impaired in alovudine-challenged *FEN1*^−/−^ cells. Interestingly, these phenotypes of *FEN1*^−/−^ cells were rescued by the loss of 53BP1, indicating a counteractive action of Fen1 and 53BP1 in the suppression of DNA damage caused by alovudine. Here, we demonstrate the previously unappreciated role of Fen1 in suppressing toxic interaction between 53BP1 and alovudine-incorporated Okazaki fragments.

## Materials and methods

### DT40 and TK6 cell culture

The DT40 cell line was obtained from Takeda Laboratory (Kyoto University). The other cell lines used are listed in Table 1. DT40 cells were cultured as previously described [22]. Briefly, the cells were cultured in DMEM/F-12 medium (GIBCO-BRL, Grand Island, NY, USA) supplemented with 10% heat-inactivated fetal bovine serum (AusgeneX, lot No. QLD 4210), 2% chicken serum (GIBCO-BRL, Grand Island, NY, USA), 50 μM mercaptoethanol (Invitrogen, CA, USA), L-glutamine (Nacalai Tesque, Japan), 50 U/ml penicillin, and 50 μg/ml streptomycin (Nacalai Tesque, Tokyo, Japan) at 39.5°C. The TK6 cell line was obtained from JCRB cell bank (https://cellbank.nibiohn.go.jp/). The other cell lines used are listed in Table 1. TK6 cells were cultured as previously described [23].

**Table 1.** DT40 and TK6 cells used in this study

Strain number	Genotype	Parental strain	References
#1	Wild-type	DT40	[24]
#6	*POLB^−/−^*	#1	[25]
#17	*ALC1^−/−^*	#1	[26]
#104	*BRCA1^−/−^*	#1	[27]
#763	*FEN1^−/−^*	#1	[28]
#1052	*53BP1^−/−^*	#1	[29]
#1133	*BRCA1^−/−^*/*53BP1^−/−^*	#1052	This study
#1175	*FEN1^−/−^/53BP1^−/−^*	#1052	This study
#T328	*XRCC1^−/−^*	#1	[30]
#52	Wild-type	TK6	[31]
#70	*ALC1^−/−^*	#52	[23]
#435	*POLB^−/−^*	#52	[23]
#1159	*FEN1^−/−^* #15	#52	[32]
#1160	*FEN1^−/−^* #17	#52	[32]
#1289	*FEN1* ^−/−^/*KU70*^−/−^	#1	This study
#1361	*FEN1^−/−^/53BP1^−/−^*	#1159	This study

### Construction of cells expressing mCherry tagged 53BP1

3xminiAID-6xFLAG tag was replaced with mCherry in the p3xmAID-6xFLAG vector [33]. Then, 2.3 kb upstream DNA sequence of the stop codon for *53BP1* gene locus was amplified using the following primer set, 5′-AAAGTCGACGCGGATCAGCATTGTCGAACAC-3′ and 5′-AAAGCTAGCACGAGGGACATAGTCATGTTTGT-3′, and cloned into the p3xmAID-6xFLAG vector. The constructed *53BP1-mCherry* targeting vector was linearized by ClaI prior to the electroporation.

### Construction of *FEN1^−/−^/53BP1^−/−^* TK6 cells

The *FEN1* gene was disrupted in TK6 cells as previously described [32]. Briefly, ∼1.2 kb of the coding sequence of Fen1 was replaced by drug resistance marker cassettes, causing the deletion of almost entire amino acid sequences of Fen1 (Supplementary Fig. S1). The loss of the Fen1 transcript was confirmed by reverse transcriptase-polymerase chain reaction using primers 5′-gagaagggagagcgagctta-3′ and 5′-agttcgagtttctgcccacc-3. The *53BP1* gene was disrupted on these cells as previously described [34].

### Construction of *BRCA1^−/−^*/*53BP1^−/−^* and *FEN1^−/−^*/*53BP1*^−/−^ DT40 cells

*BRCA1* and *53BP1* genes were disrupted as previously described [27–29].

### Cellular sensitivity assay

A liquid culture cell survival assay was performed as previously described to measure cellular sensitivity to alovudine [26, 35]. Briefly, the DT40 and TK6 cells were diluted in medium (0.5 × 10^4^ cells/ml) and dispensed into 96-well plates (80 μl/well). A total of 80 μl of alovudine diluted with culturing medium was added and mixed, and the cells were cultured for 48 h for DT40 cells and 72 h for TK6 cells before being transferred to 96-well plates (100 μl/well). ATP levels were measured using the CellTiter-Glo Cell Viability Assay kit (Promega, MA, WI, USA) according to the manufacturer’s instructions. Luminescence was measured using a Fluoroskan Ascent FL Microplate Fluorometer and Luminometer (Thermo Fisher Scientific Inc., Waltham, MA, USA).

### Chromosomal aberration analysis

Mitotic chromosome spreads were prepared and analyzed as described previously [36]. The DT40 cells were arrested in the M phase by treatment with colcemid (Thermo Fisher Scientific Inc., Waltham, MA, USA) at a concentration of 0.1 μg/ml for 2 h. The cells were pelleted, resuspended in 75 mM KCl (1.0 ml), and incubated for 13 min at room temperature. Then, the cells were fixed in a freshly prepared 3:1 mixture of methanol and acetic acid (200 μl; Carnoy’s solution). After pelleting, the cells were resuspended in 700 μl of Carnoy’s solution and dispensed onto cold glass slides (∼10^6^ cells/slide), followed by air-drying. The slides were stained with 5% HARLECO Giemsa Stain solution (Nacalai Tesque) for 10 min, rinsed with water and acetone, and dried at 20°C. The slides were examined under an ECLIPSE-Ni microscope (NIKON, Tokyo, Japan) at 1000 × magnification. The chromosomes within each mitotic cell were scored.

### Immunofluorescent visualization of subnuclear γH2AX

The experimental conditions for the immunocytochemical analysis have been described previously [37]. Briefly, following treatment of the DT40 cells with alovudine for 5 h at 39.5°C, the cells were collected on glass slides using a Cytospin apparatus (Shandon, Pittsburgh, PA, USA), fixed with 4% formaldehyde for 10 min at room temperature, permeabilized with 0.5% Triton X-100, rinsed twice in phosphate-buffered saline (PBS), and blocked using PBS/0.5% bovine serum albumin (BSA). Then, the cells were incubated with an anti-γ-H2AX antibody (Millipore; JBW301, MA, USA) diluted 1/400 in PBS/0.5% BSA for 1 h at room temperature and washed thrice with PBS. Next, the cells were incubated with Alexa Fluor 488 goat anti-rabbit IgG antibody (Invitrogen, diluted 1/200) in PBS/0.5% BSA for 1 h at room temperature and rinsed thrice in PBS, followed by counterstaining with 4′,6′-diamidino-2-phenylindole (DAPI). The images were captured using a BZ-X810 fluorescence microscope (Keyence, Tokyo, Japan).

### DNA fiber assays

DNA fiber assays were performed as previously described [26], with slight modifications to the labeling method used for the replication tracts. Briefly, the cells were sequentially labeled for 15 min with 25 μM 5-chloro-2′-deoxyuridine (CldU) and 250 μM 5-iodo-2′-deoxyuridine (IdU). Fiber length was measured using ImageJ software (https://imagej.nih.gov/ij/docs/faqs.html), and the CldU/IdU ratio was calculated. Measurements were recorded from areas of the slides with untangled DNA fibers to prevent the possibility of recording labeled patches from tangled fiber bundles. Images were captured using a BZ-X810 fluorescence microscope (Keyence).

### Cell cycle analysis by flow cytometry

Cell cycle analysis was performed as previously described [2]. Briefly, the cells were fixed in 70% ethanol, stained with propidium iodide in the presence of RNase A, and analyzed using BD Accuri^TM^ C6 (Becton Dickinson, NJ, USA).

### Alkaline and neutral comet assay

Chicken DT40 cells were treated with 100 μM of alovudine for 6 h at 39.5°C, and the tail DNA moments [38] that reflect the induced damages were measured. Alkaline and neutral comet (single-cell gel electrophoresis) assays were performed as described previously [23]. For the comet assay, the cells (5 × 10^4^ cells/slide) were resuspended in 70 μl of 1% low-melting agarose (Sigma–Aldrich, WI, USA) in PBS and spread onto microscopy slides coated with 1% agarose (Bio-Rad, CA, USA). The cells were lysed in the lysis solution [2.5 M NaCl, 100 mM Na_2_ethylenediaminetetraacetic acid (EDTA), 10 mM Tris–base, 5 M NaOH to maintain pH at 10; 1% Triton X-100 and 0.5% N-lauroylsarcosine sodium salt were added prior] at 4°C for 2 h. Each slide was washed thrice for 5 min in PBS and cold running buffer (300 mM NaOAc, 100 mM Tris–HCl, pH was adjusted to 8 using HCl) for alkaline and neutral assay, respectively. Then, the alkaline comet slides were placed in a tank with cold running buffer (300 mM NaOH, 1 mM Na_2_ EDTA, pH was adjusted to 13 using HCl) for 40 min before electrophoresis for 50 min at 25 V (current flow was 0.52–0.6A). The neutral comet slides were run at 25 V for 60 min. Comet Analysis System OpenComet [39] was used to quantify tail DNA moments in DT40 cells and at least 300 cells (from three experiments) were scored per sample.

### BrdU comet assay

To label S-phase cells and measure breaks in nascent strands during the maturation of DNA replication intermediates, cells were pulse-labeled with 100 μM BrdU for 30 min. Afterword, the cells were released in drug-free medium for 3 h (for measuring breaks in nascent strands). Then, alkaline comet assay was performed. In this case, after electrophoresis, slides were washed three times for 5 min each by layering with neutralization buffer (0.4 M Tris–HCl, pH 7.4). Slides were then immersed in 70% ethanol for 5 min, dried at 37°C for 10 min, washed three times for 10 min each in PBS, and blocked with blocking buffer (0.1% Tween 20, 3% BSA, in PBS) for 30 min by horizontal layering at room temperature. After blocking, slides were incubated with the mouse monoclonal anti-BrdU antibodies (cat. no. 347 580; Beckton Dickinson; 1:50) overnight at 4°C in a humid chamber, then washed three times for 10 min each in TBST and incubated with secondary antibodies (anti-mouse Alexa Fluor 647, cat. no. A21235; Invitrogen; 1:500) in blocking buffer for 1 h at room temperature in the dark. Thereafter, slides were washed three times for 10 min in TBST, mounted with PermaFlour (Vector Laboratories, CA, USA) and covered with coverslips. OpenComet [39] was used to quantify the tail moment and >150 cells (from three experiments) were scored per sample.

### Immunofluorescent visualization of subnuclear γH2AX and 53BP1 (mCherry)

The experimental conditions for the immunocytochemical analysis have been described previously [32]. Briefly, following treatment of the DT40 cells with alovudine for 0–5 h at 39.5°C, the cells were collected on glass slides using a Cytospin apparatus (Shandon, Pittsburgh, PA, USA), fixed with 4% formaldehyde and 0.1% Triton X-100 for 10 min at room temperature, permeabilized with 0.5% Triton X-100, rinsed twice in PBS, and blocked using 0.5% BSA in 1× PBS. Then, the cells were incubated with an anti-γ-H2AX mouse antibody (Millipore; JBW301, MA, USA, diluted 1:400) and anti-mCherry rabbit antibody (α-RFP, MBL, IL, USA, diluted 1:500) diluted in PBS/0.5% BSA for 1 h at room temperature and washed thrice with PBS. Next, the cells were incubated with Alexa Fluor 488 anti-mouse IgG antibody (Invitrogen, diluted 1/200) and Cy3 anti-rabbit IgG antibody (Invitrogen, diluted 1/200) in PBS/0.5% BSA for 1 h at room temperature and rinsed thrice in PBS, followed by counterstaining with DAPI. The images were captured using a BZ-X810 fluorescence microscope (Keyence, Tokyo, Japan).

### Immunofluorescent visualization of subnuclear 53BP1 (mCherry) and EdU foci

The experimental conditions for the immunocytochemical analysis have been described previously [40]. Briefly, following treatment of the DT40 cells with alovudine for 5 h at 39.5°C, the cells were washed and incubated for 30 min at 39.5°C, followed by EdU treatment for 15 min. The cells were collected on glass slides using a Cytospin apparatus (Shandon, Pittsburgh, PA, USA), fixed with 4% formaldehyde and 0.1% Triton X-100 for 10 min at room temperature, permeabilized with 0.5% Triton X-100, rinsed twice in PBS, and blocked using 0.5% BSA in 1× PBS. Then, the cells were incubated with an anti-mCherry rabbit antibody (α-RFP, MBL, IL, USA, diluted 1:500) diluted in PBS/0.5% BSA for 1 h at room temperature and washed thrice with PBS. Next, the cells were incubated with Cy3 anti-rabbit IgG antibody (Invitrogen, diluted 1/200) in PBS/0.5% BSA for 1 h at room temperature and rinsed thrice in PBS. Further, the cells were incubated with Click iT reaction buffer (Thermo Fisher Scientific Inc., MA, USA) for 1h and rinsed thrice in PBS followed by counterstaining with DAPI. The images were captured using a BZ-X810 fluorescence microscope (Keyence, Tokyo, Japan).

### Measurement of colocalization

Colocalization analysis experiments were performed according to the previously described method [41], using the Coloc2 plugin of the Fiji software [42]. An average of 50 individual cells from three to five independent representative images per condition captured from one representative experiment was used to calculate Pearson’s colocalization coefficient with automatic Costes thresholding [43].

### Measurement of sister chromatid exchanges

Sister chromatid exchange (SCE) was determined as follows: DT40 cells were incubated in a medium containing alovudine (100 μM) and 5-bromo-2′-deoxy-uridine (BrdU) (10 μM) at 39.5°C for 16 h, which corresponds to two cell cycle periods for these cells. For the last 2.5 h of the incubation period, the cells were treated with colcemid (0.1 μg/ml) to enrich mitotic cells. Next, the cells were pelleted through centrifugation (1200 rpm for 5 min), resuspended in 75 mM KCl (10 ml) for 13 min at 20°C, and fixed in freshly prepared Carnoy’s solution (10 ml). The pelleted cells were resuspended in Carnoy’s solution (0.4 ml), placed onto clean glass slides (Matsunami Glass, Osaka, Japan), and air-dried. The dried slides were incubated with Hoechst 33 258 nuclei acid stain (10 μg/ml) in phosphate buffer (pH 6.8) for 20 min and rinsed with McIlvaine buffer (164 mM Na_2_HPO_4_ and 16 mM citric acid; pH 7.0). Next, the slides were irradiated using black light (λ = 352 nm) for 25 min and incubated in saline–sodium citrate solution (0.15 M NaCl and 0.015 M sodium citrate) at 58°C for 20 min, followed by staining with 5% HARLECO Giemsa Stain Solution (Nacalai Tesque, Kyoto, Japan) for 10 min. The images were captured using a BZ-X810 fluorescence microscope (Keyence).

## Results

### The critical role of Fen1 in the cellular tolerance to alovudine

**Figure 1.**
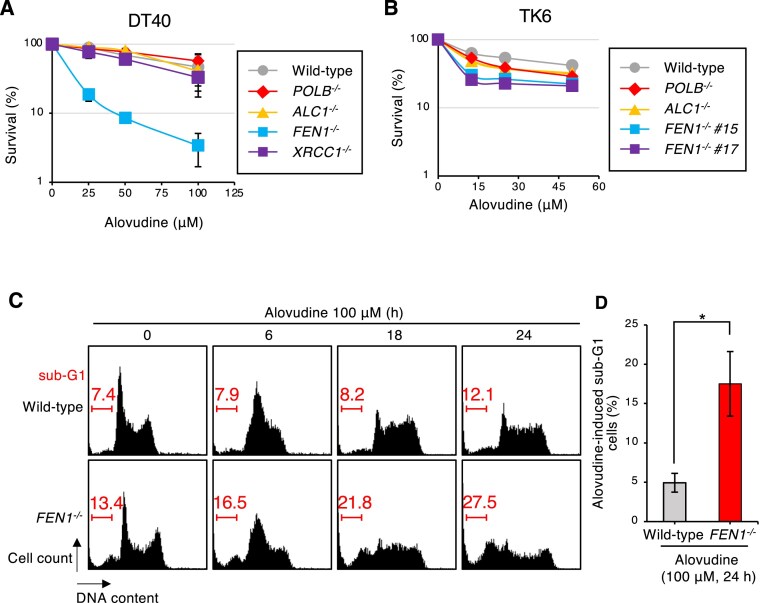
The critical role of Fen1 in the cellular tolerance to alovudine. (**A, B**) Indicated DT40 cells (A) and TK6 cells (B) were assessed for sensitivity to alovudine. DT40 cells and TK6 were cultured in the presence of the indicated concentration of alovudine for 48 and 72 h, respectively. The dose of alovudine is displayed on the *x*-axis on a linear scale, and the percentage of cell survival is displayed on the *y*-axis on a logarithmic scale. Error bars represent the standard deviation (SD) from three (two for TK-6 cells) independent experiments. (**C**) Cells were treated with 100 μM of alovudine for the indicated period. The histogram presents the cell cycle distribution. The DNA content (stained using propidium iodide) is displayed on the *x*-axis on a linear scale and the number of cells counted is presented on the *y*-axis. The left bars represent a percentage of cells falling in the sub-G1 (dead cell) fraction. (**D**) Quantification of the percentage of alovudine-induced sub-G1 fraction. Error bars represent the SD from six independent experiments. The *P*-value was calculated using paired *t*-test. *P < .05.

Our recent screening to identify factors involved in the cellular tolerance to alovudine identified BRCA1 as a dominant factor required for maintaining cellular survival upon alovudine challenge [7]. In this screening, we also identified Fen1 as a critical factor for the cellular tolerance to alovudine [7]. However, the role played by Fen1 in suppressing DNA damage caused by alovudine has not been clarified. As shown in Fig 1A, the loss of Fen1 critically sensitized the chicken DT40 cells to alovudine (Fig 1A). We also found that *FEN1*^−/−^ cells generated from human TK6 cells showed hypersensitivity to alovudine (Fig. 1B), indicating a conserved role of Fen1 in suppressing cell death upon alovudine challenge. Consistent with this Fen1’s role in the alovudine tolerance, we observed that alovudine treatment induced more sub-G1 population (dead cell fraction) in *FEN1*^−/−^ cells than in wild-type cells after transient cell cycle arrest at mid-S phase (Fig. 1C and D). To investigate how Fen1 contributes to the cellular tolerance to alovudine, we analyzed the contribution of BER factors, such as polymerase β, ALC1, or XRCC1 to the cellular tolerance to alovudine (Fig. 1A and B). We found these mutants and wild-type cells exhibited indistinguishable sensitivity to alovudine, and only *FEN1*^−/−^ cells showed significantly higher hypersensitivity to alovudine than did wild-type cells (Fig. 1A and B). These data suggest that Fen1 plays an important role in the cellular tolerance to alovudine independently from its BER function.

### The loss of 53BP1 restores alovudine hypersensitivity in *FEN1*^−/−^ cells

**Figure 2.**
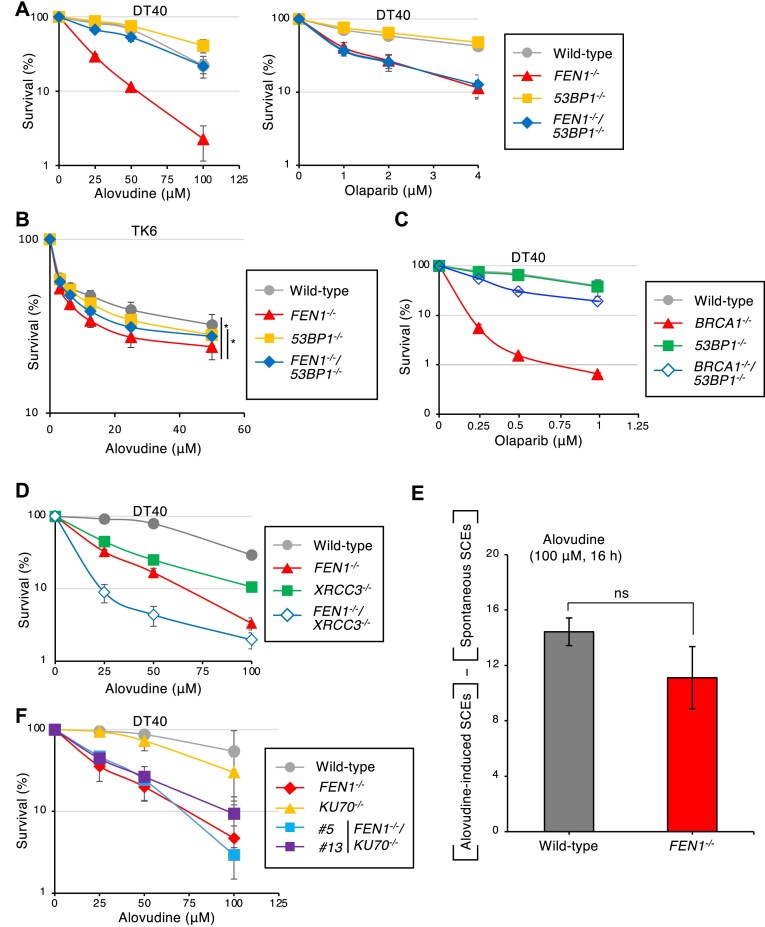
The *53BP1* gene disruption restores alovudine hypersensitivity in *FEN1*^−/−^ cells. (**A**) Indicated DT40 cells were assessed for sensitivity to alovudine or olaparib. DT40 cells were cultured as in Fig. 1A. The dose of the indicated drug is displayed on the *x*-axis on a linear scale, and the percentage of cell survival is displayed on the *y*-axis on a logarithmic scale. Error bars represent the SD from three independent experiments. (**B**) Indicated TK6 cells were assessed for sensitivity to alovudine. TK6 cells were cultured as described in the ”Materials and methods" section. Error bars represent the SD from three independent experiments. The *P*-value was calculated using paired *t*-test. *P < .05 (**C, D, F**) Indicated DT40 cells were assessed for sensitivity to alovudine or olaparib. DT40 cells were cultured as in Fig. 1A. The dose of the indicated drug is displayed on the *x*-axis on a linear scale, and the percentage of cell survival is displayed on the *y*-axis on a logarithmic scale. Error bars represent the SD from three independent experiments. (**E**) Analysis of SCEs in the indicated DT40 cells treated with alovudine (100 μM) and BrdU for 16 h. Numbers of SCEs were scored for 50 mitotic nuclei per experiment. The number of SCEs in untreated cells was subtracted from that in alovudine-treated cells. The bar graph represents the means ± SD from three independent experiments. The *P*-value was calculated using Student’s *t*-test. ns, not significant.

Since we previously demonstrated that the alovudine-hypersensitivity of *BRCA1*^−/−^ cells was rescued by the loss of 53BP1 [7], we next examined the relationship between Fen1 and 53BP1. We found that the loss of 53BP1 has little impact on the cellular tolerance to alovudine (Fig. 2A). Strikingly, we found that the loss of 53BP1 in *FEN1*^−/−^ cells restored the hypersensitivity of alovudine (Fig. 2A). Similarly, *FEN1*^−/−^ cells’ alovudine-hypersensitivity was rescued by the loss of 53BP1 in human TK6 cells (Fig. 2B), indicating the relationship between Fen1 and 53BP1 is conserved between chicken and human. Since the restoration of alovudine-hypersensitivity in *BRCA1*^−/−^ was associated with the restoration of HR function by the loss of 53BP1 [7], we next examined the sensitivity of these cells to the PARP inhibitor olaparib, which induces DNA damage requiring HR for repair [44] (Fig. 2A). We found that *FEN1*^−/−^ and *FEN1*^−/−^/*53BP1*^−/−^ cells exhibited similarly higher sensitivity to olaparib than wild-type cells (Fig. 2A), indicating that the restoration of cellular survival to alovudine in *FEN1*^−/−^/*53BP1*^−/−^ cells is caused by a mechanism distinctly different from that in *BRCA1*^−/−^/*53BP1*^−/−^ cells, since the loss of 53BP1 in *BRCA1*^−/−^ cells rescued olaparib-hypersensitivity of BRCA1 deficient cells (Fig. 2C). Moreover, the loss of Fen1 in HR defective *XRCC3*^−/−^ cells further sensitized cells to alovudine (Fig. 2D), suggesting that Fen1 promotes alovudine tolerance independently from HR. To analyze the functionality of HR in *FEN1*^−/−^ cells, we measured the number of alovudine-induced SCEs, a hallmark of HR events [45]. We found that *FEN1*^−/−^ cells were proficient to induce SCEs upon alovudine treatment, indicating that Fen1 contributes to alovudine tolerance through functions other than HR (Fig. 2E). We next asked if the loss of functionality of the nonhomologous end joining (NHEJ) rescues *FEN1*^−/−^ cells’ alovudine-hypersensitivity since 53BP1 is involved in the NHEJ [46]. To this end, we disrupted a critical gene for NHEJ, *KU70*, in *FEN1*^−/−^ DT40 cells. We found that the loss of KU70 in Fen1 deficient cells has no impact on their cellular tolerance to alovudine (Fig. 2F), suggesting that 53BP1 is involved in the cellular tolerance to alovudine independent of its NHEJ function.

### Fen1 promotes cellular tolerance to alovudine by counteracting toxic effects of 53BP1 upon alovudine treatment

**Figure 3.**
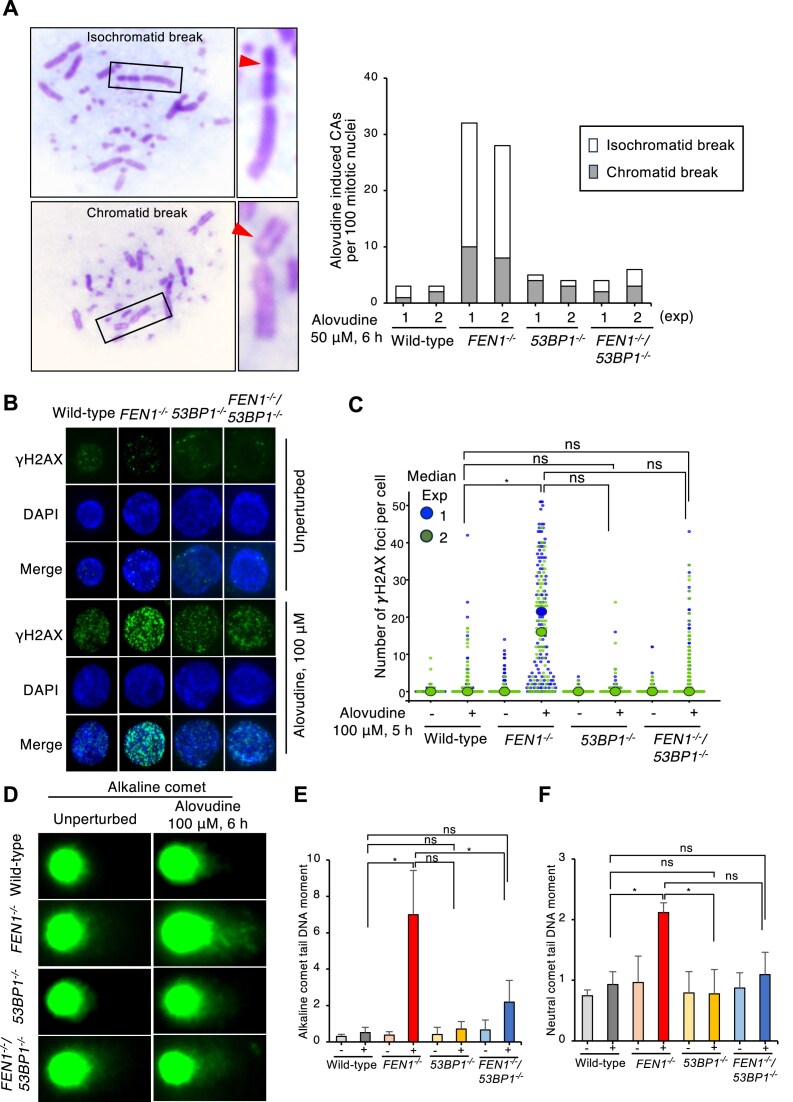
Fen1 promotes cellular tolerance to alovudine by counteracting 53BP1. (**A**) Representative images showing chromatid breaks and isochromatid breaks (arrowheads). Number of CAs/100 mitotic nuclei in the indicated genotypes. Error bars represent SD. At least 75 mitotic cells were counted for each cell line and experiment. (**B**) Representative fluorescence microscopic images of γH2AX foci in the indicated cell lines at 5 h after exposure to 100 μM alovudine. All statistical analyses were performed using Student’s *t*-test. *P < .05. (C) The scatter plots show the number of γH2AX foci per cells. At least 100 cells were scored per analysis. The experiments were repeated twice. The plots were generated using SuperPlotsOfData (https://huygens.science.uva.nl/SuperPlotsOfData/). The *P*-value was calculated using Student’s *t*-test. *P < .05, ns, not significant; *P* > .05. (**D**) Representative fluorescence microscopic images of the alkaline comet in the indicated cell lines at 6 h of alovudine exposure. (**E, F**) DNA breaks in genomic DNA quantified by alkaline comet assay (**E**) and neutral comet assays (**F**) in DT40 cells with the indicated genotypes following incubation (6 h) with alovudine (100 μM). Comet tail moments (an arbitrary unit of DNA breaks) were scored following the staining of genomic DNA with SybrGold. We analyzed >60 nuclei and medians of comet tail moment. The bar graph represents the mean and SD of medians from three independent experiments (n = 3). Dot plots showing all measured tail moments in individual nuclei are shown in Supplementary Fig. S2. The *P*-value was calculated using Student’s *t*-test. *P < .05, ns, not significant; *P* > .05.

We found unexpected restoration of alovudine-hypersensitivity of *FEN1*^−/−^ cells by the loss of 53BP1 (Fig. 2); we thus next asked if the loss of 53BP1 suppresses DNA damage induced by alovudine in *FEN1*^−/−^ cells. We found that *FEN1*^−/−^ cells exhibited an augmented number of chromosomal aberrations (CAs) upon alovudine treatment compared to wild-type cells, and the loss of 53BP1 significantly reduced the alovudine-induced CAs in *FEN1*^−/−^ cells (Fig. 3A). Similarly, we found that *FEN1*^−/−^ cells exhibited an augmented γH2AX signal (a maker for DNA damage) upon alovudine treatment compared to wild-type cells, and the loss of 53BP1 in *FEN1^−/−^* cells reduced the alovudine-induced γH2AX signal in *FEN1^−/−^* cells (Fig. 3B and C). These data suggest that 53BP1 induces DNA damage upon alovudine treatment, which is suppressed by Fen1. Next, we employed alkaline comet assay and neutral comet assay to determine the number of single-strand breaks (SSBs) and DSBs, respectively (Fig. 3D–F). These tests demonstrated that the number of alovudine-induced DNA damage is significantly increased in *FEN1*^−/−^ cells, but the augmented alovudine-induced DNA damage in Fen1 deficient cells was critically reduced by the loss of 53BP1 (Fig. 3D–F and Supplementary Fig. S2A and B). Taken together, these data indicate that Fen1 promotes cellular tolerance to alovudine by counteracting toxic effects of 53BP1 upon alovudine treatment.

### Fen1 maintains replication fork progression in the presence of alovudine

**Figure 4.**
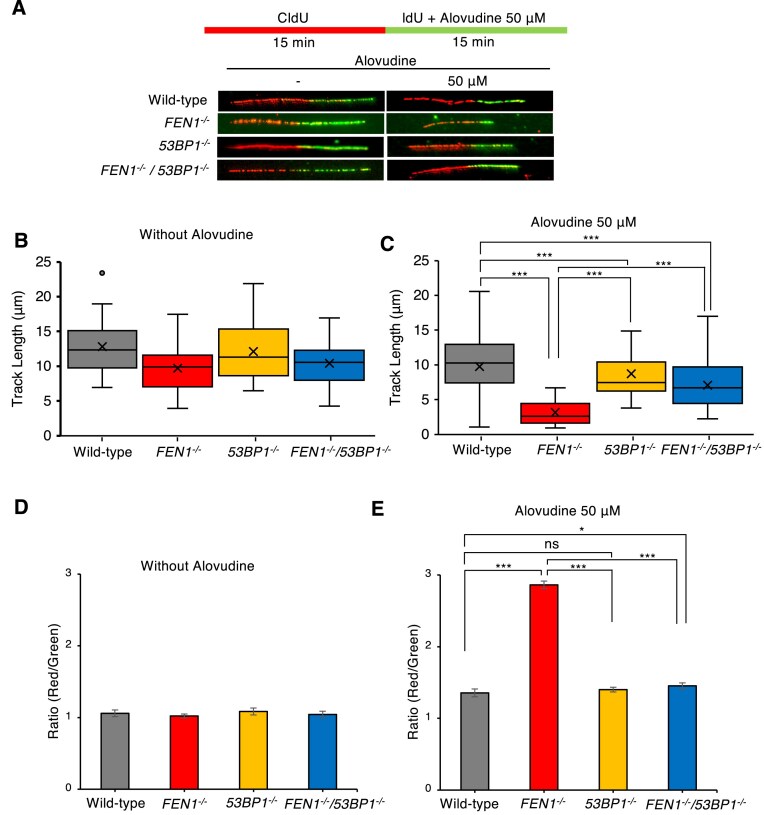
Important role of Fen1 in the maintenance of the replication fork progression in the presence of alovudine. (**A**) Schematic representation of the protocol to monitor fork speed. Indicated DT40 cells were sequentially pulse-labeled with CldU and IdU for 15 min each. Cells were exposed in 50 μM alovudine in the second labeling. Representative images showing DNA fibers from DT40 cells. (**B, C**) Lengths of the CldU and IdU tracks without (B) or with (C) alovudine treatment were measured for at least 50 forks. (**D, E**) The medians of CldU/IdU ratio were obtained from three independent analyses and means and SDs of ratios were presented. All statistical analyses were performed by two two-tailed Student’s *t*-test. ns, not significant. *P < .05, ***P < .001.

Having established the genetic interaction between *FEN1*^−/−^ and *53BP1*^−/−^ in suppressing DNA damage upon alovudine and ultimately in the cellular tolerance to alovudine (Figs 2 and 3), we next investigated the effect of Fen1 and 53BP1 in the kinetics of replication fork progression in the presence of alovudine, since Fen1 is a central factor for Okazaki fragment maturation [13] and 53BP1 also plays a critical role in this process by recruiting Dna2 to nascent DNAs [16]. To investigate the impact of alovudine on replication fork progression, we employed DNA fiber assay [47] to measure the kinetics of replication fork progression. The cells were sequentially pulse-labeled with CldU and IdU for 15 min each and exposed to alovudine during IdU labeling. As a result, replicated tracks were identified before (CldU, red) and after (IdU, green) alovudine treatment (Fig. 4). We found that the replication fork progression was linearly reduced upon alovudine exposure in dose dependent manner at the ranges (0–200 μM) for wild-type DT40 cells [7] and (0–100 μM) for wild-type TK6 cells (Supplementary Fig. S3A). For further experiments, we used 50 or 25 μM of alovudine to evaluate the effect of alovudine on replication kinetics in DT40 or TK6 cells, respectively (Fig. 4A and Supplementary Fig. S3B). We found that alovudine treatment critically slowed replication fork progression in *FEN1*^−/−^ cells compared to wild-type cells (Fig. 4B–E), suggesting that Fen1 suppresses the incorporation of alovudine into nascent DNA to mitigate the chain-terminating effect of alovudine. This was also the case in human TK6 cells since we observed a significant reduction of the replication track lengths in alovudine-treated duration in *FEN1*^−/−^ TK6 cells (Supplementary Fig. S3C–E). These data indicate the conserved role of Fen1 in the suppression of chain-terminating effects of alovudine. Remarkably, the loss of 53BP1 rescued impaired alovudine-induced replication fork progression in *FEN*^−/−^ DT40 cells (Fig. 4B–E). Collectively, these data revealed the importance of Fen1 in maintaining DNA replication, in which Fen1 counteracts the toxic effect of 53BP1 on alovudine-challenged nascent DNA, thereby suppressing the chain-terminating effect.

### The unimportant role of Fen1 in the maintenance of replication fork in the alovudine-incorporated template

Our recent study revealed that alovudine also exerts its cytotoxic effects via serving as DNA damage on the template strand in addition to its chain-terminating activity [7]. We next investigated the role of Fen1 in the maintenance of replication on the alovudine-damaged template. To this end, we exposed the cells to alovudine for 12 h (corresponding to 1.5 cell cycle length of DT40 cells) followed by a release for 30 min. Then, cells were sequentially pulse-labelled with CldU (red) and IdU (green) for 15 min each (Supplementary Fig. S4A). We measured the total length of CldU (red) and IdU (green) strands and calculated the track length. We found that the replication fork progression was not significantly reduced in *FEN1*^−/−^ cells compared with that in wild-type cells (Supplementary Fig. S4B–D). These data indicate that Fen1 does not play a role in the maintenance of replication fork progression on the alovudine-incorporated template.

### The critical role of Fen1 in the suppression of the toxic effect of 53BP1 on the maturation of alovudine-incorporated Okazaki fragment

**Figure 5.**
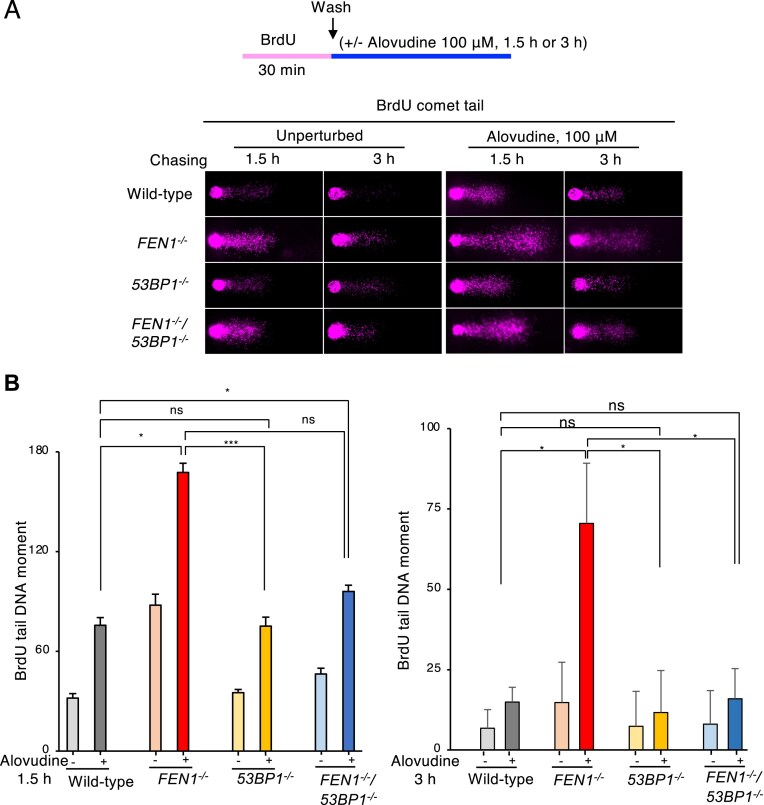
Fen1 suppresses the toxic effect of 53BP1 on the maturation of alovudine-incorporated Okazaki fragment. (**A**) Schematic representation of the experimental protocol of BrdU comet assay. Representative fluorescence microscopic comet images of the indicated cell lines with or without exposure of 100 μM alovudine. (**B**) BrdU comet tail moments were quantified in the indicated cells following incubation with alovudine (100 μM). BrdU comet tail moments were scored by staining with anti-BrdU antibodies following a 0.5 h BrdU pulse label and subsequent 1.5 or 3 h chase. For each sample, the tail moments of more than 40 individual nuclei were obtained per experiment and the bar graph represents the mean and SD of medians of tail moments obtained from three independent experiments. All statistical analyses were performed by two two-tailed Student’s *t*-test. *P < .05. ***P < .01. ns: not significant.

Having established the role of Fen1 in suppressing the toxic effect of 53BP1 in maintaining replication fork progression in the presence of alovudine, we next asked if Fen1 counteracts 53BP1-mediated avoidance of the maturation of the Okazaki fragment in the presence of alovudine. The defects in Okazaki fragment maturation result in the accumulation of unligated Okazaki fragments, a major source of SSBs [48]. To monitor the kinetics of the Okazaki fragment maturation, we measured the number of SSBs in the nascent DNA. We pulse labeled wild-type and *FEN1*^−/−^ cells with BrdU for 30 min and after washing the cells we chased for 0, 1.5, 3, and 6 h with and without alovudine followed by alkaline comet assay (Supplementary Fig. S5A and B). With this assay, the kinetics of Okazaki fragment maturation is detectable in the alkaline comet assay as BrdU stained comet tails [49]. We found that *FEN1^−/−^* cells showed slightly augmented BrdU comet tail moment compared to wild-type cells, indicating the delayed Okazaki fragment maturation in *FEN1^−/−^* cells (Supplementary Fig. S5A). Moreover, BrdU comet tail moment was critically increased in *FEN1^−/−^* cells when cells were treated with alovudine (Supplementary Fig. S5B). Since we found *FEN1^−/−^* cells showed considerable BrdU comet tail at 1.5 and 3 h of chase period when wild-type cells completed Okazaki fragment maturation even in the presence of alovudine, we chose 1.5 and 3 h as the chase time to compare wild-type, *FEN1^−/−^*, *53BP1^−/−^*, and *FEN1^−/−^*/*53BP1^−/−^*cells (Fig. 5A). We found that the Okazaki fragment maturation was significantly slowed in *FEN1*^−/−^ cells upon alovudine exposure compared to wild-type cells (Fig. 5B). Strikingly, the loss of 53BP1 restored the *FEN1^−/−^* cells’ defects in Okazaki fragment maturation in the presence of alovudine (Fig. 5B). These data indicate that Fen1 counteracts the inhibitory effects of 53BP1 on the maturation of the Okazaki fragment in the presence of alovudine during lagging strand synthesis.

### Alovudine induces 53BP1 foci formation in *FEN1^−/−^* DT40 cells

**Figure 6.**
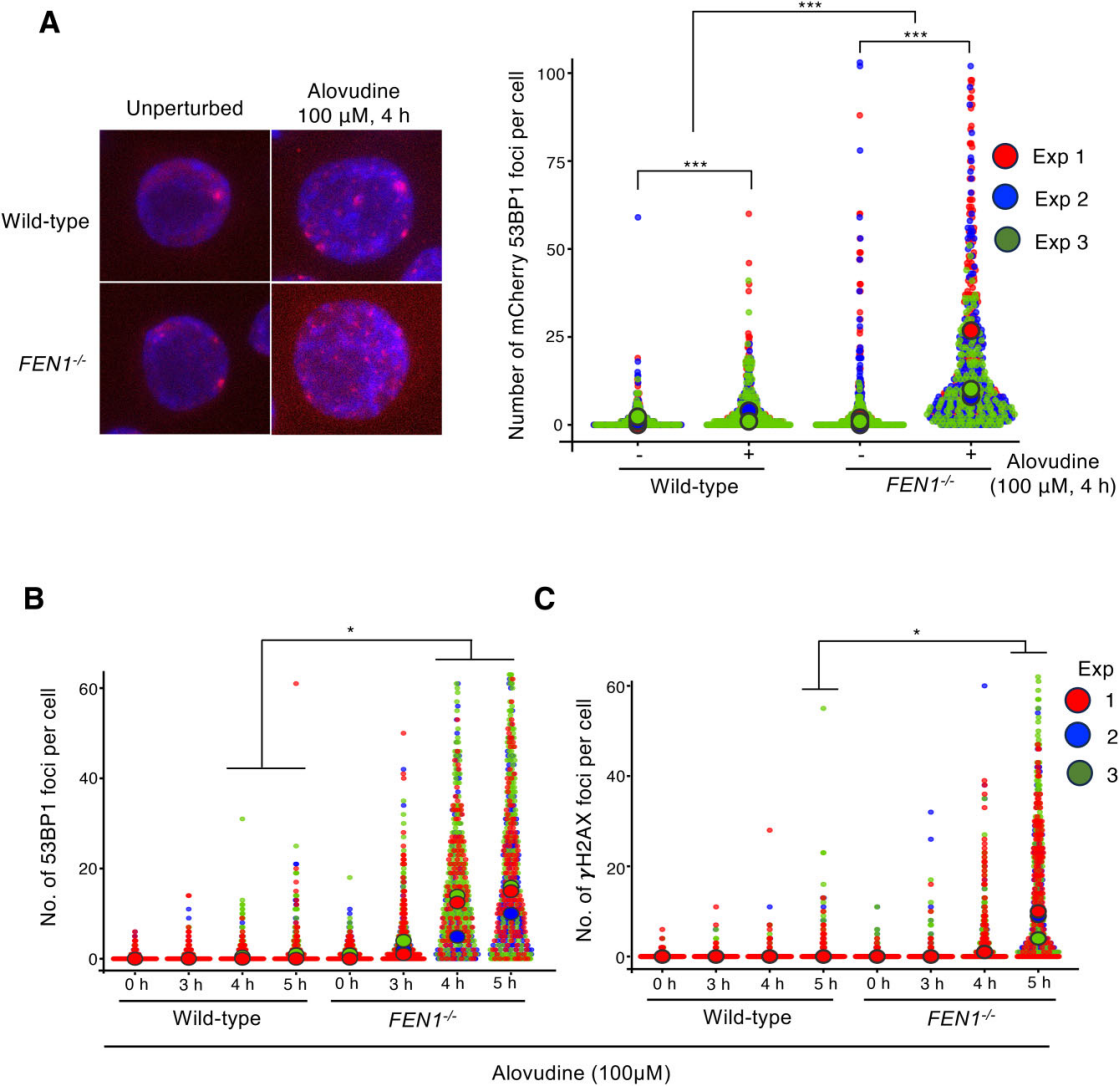
Alovudine induces augmented 53BP1 and γH2AX foci in the absence of Fen1. (**A**) Indicated DT40 cells were treated with alovudine (100 μM) for 4 h and 53BP1-mCherry signal was assessed under the luminescence microscope. The number of 53BP1-mCherry foci was measured for >65 cells. Plots were generated using SuperPlotsOfData. For each sample, scatter plots represent number of 53BP1 foci per cell for >50 individual cells and the circles represent the medians. All statistical analyses were performed by two two-tailed Student’s *t*-test. ***P < .001. (**B, C**) Indicated cells were treated with alovudine for 0, 3, 4, and 5 h. The number of 53BP1 (B) and γH2AX (C) foci was measured independently three times. All statistical analyses were performed by two two-tailed Student’s *t*-test. *P < .05.

To investigate the interfering effect of 53BP1 in the maturation of alovudine-incorporated Okazaki fragments, we analyzed the number of 53BP1 foci induced by alovudine. The alovudine treatment induced a significantly higher level of 53BP1 foci in *FEN1^−/−^* DT40 cells than in wild-type cells (Fig. 6A and Supplementary Fig. S6), indicating that Fen1 suppresses alovudine-induced 53BP1 subnuclear foci formation. Collectively, these data suggest that Fen1 prevents the interaction of 53BP1 with alovudine-incorporated nascent DNA to prevent 53BP1’s inhibitory effects on the maturation of the Okazaki fragment in the presence of alovudine. Should this be the case, 53BP1-mediated avoidance of the Okazaki fragment maturation in *FEN1^−/−^* cells might induce DNA damage. This was indeed the case since *FEN1^−/−^* cells exhibited a similarly augmented number of γH2AX and 53BP1 foci and their localization was significantly overlapped at 5 h after alovudine treatment (Fig. 6B and C, and Supplementary Fig. S6). We also observed the accumulation of 53BP1 foci preceded that of γH2AX, implying that 53BP1 accumulation is the cause, but not the consequence of DNA damage (Fig. 6B and C). Collectively, our data indicate the influential role of Fen1 to suppress the toxic effect of alovudine-induced 53BP1 in the Okazaki fragment maturation in chicken DT40 cells.

**Figure 7.**
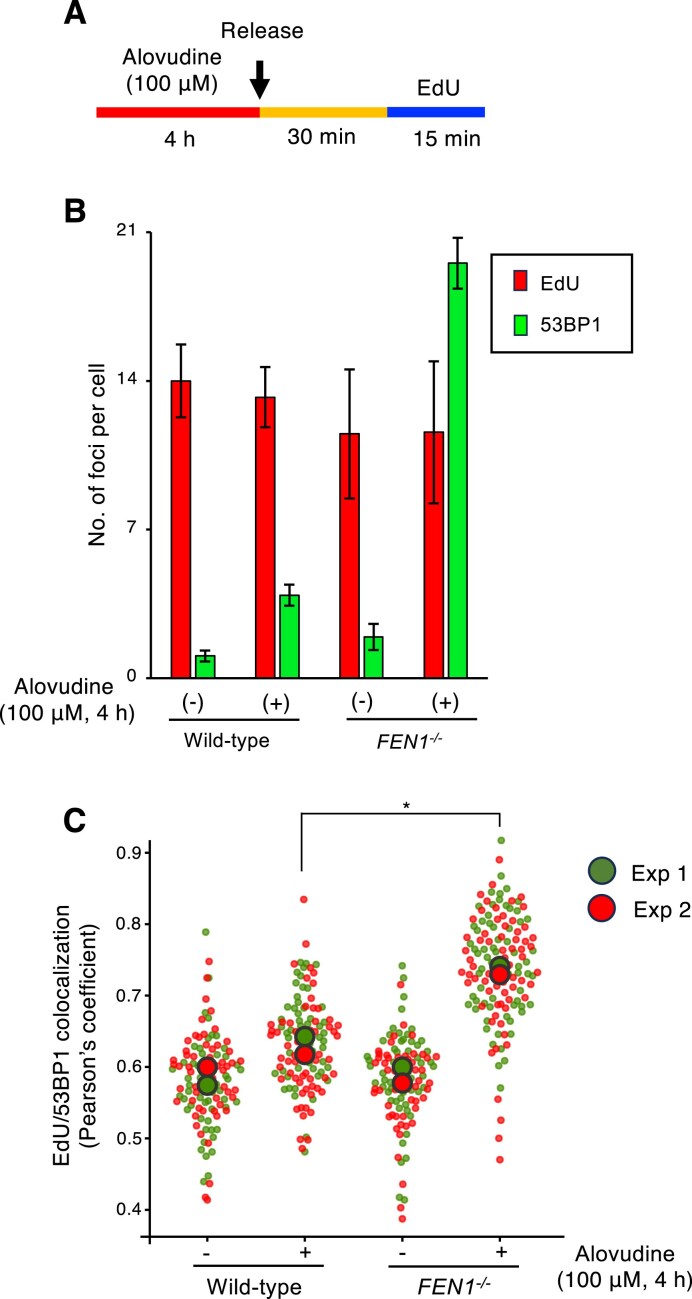
Fen1 suppresses the accumulation of 53BP1 on alovudine-incorporated nascent DNA. (**A**) Schematic representation of the experimental protocol to detect the overlap of 53BP1 foci and nascent DNA synthesis sites. Indicated DT40 cells were treated with alovudine (100 μM) for 4 h and released in a drug-free medium for 30 min. Cells were then pulse labeled with EdU for 15 min. (**B**) The number of 53BP1 foci and EdU foci was counted for at least 90 nuclei. Representative images were shown in Supplementary Fig. S7. The experiment was repeated two times. The bar graph represents the average and SD from two independent experiments. (**C**) The degree of overlap between 53BP1 foci and nascent DNA synthesis sites (EdU foci) was calculated as Pearson’s correlation using Fiji software [42]. Plots were generated using SuperPlotsOfData. For each sample, scatter plots represent Pearson’s correlation per cell for >50 individual cells and the circles represent the medians. All statistical analyses were performed by two two-tailed Student’s *t*-test. *P < .05.

To further confirm if Fen1 indeed suppresses the accumulation of the 53BP1 foci at alovudine-incorporated nascent DNA, we examined the sites of nascent DNA synthesis and 53BP1 foci. To this end, we pulse-treated the cells with alovudine for 4 h and released them in a drug-free medium to allow replication, and then the sites of nascent DNA synthesis were labeled with EdU (Fig. 7A and Supplementary Fig. S7). We found a similar number of replication foci in wild-type and *FEN1^−/−^* cells irrespective of alovudine treatment, whilst Fen1 deficient cells exhibited a higher number of 53BP1 foci upon alovudine compared to wild-type cells (Fig. 7B). Moreover, we found significant overlap between 53BP1 foci and nascent DNA sites (Fig. 7C). These data indicate that Fen1 indeed suppresses the accumulation of 53BP1 on alovudine-incorporated nascent DNA.

## Discussion

In this study, we explored mechanisms underlying the genotoxic effects of alovudine and the role of Fen1 to suppress DNA damage induced by alovudine. We found that only Fen1, but not other BER factors such as polymerase β, ALC1, or XRCC1 play a critical role in the cellular tolerance to alovudine (Figs 1 and 2). Fen1 maintains replication fork progression in the presence of alovudine (Fig. 3 and Supplementary Fig. S3) and suppresses DNA damages (Fig. 2). Remarkably, the loss of 53BP1 rescued all phenotype of *FEN1^−/−^* cells (Figs 2–5), suggesting that Fen1 counteracts the toxic effects of 53BP1 on avoidance of replication fork arrests and DNA damage caused by alovudine. This was indeed the case since we observed that delayed maturation of the alovudine-incorporated Okazaki fragments in *FEN1^−/−^* cells was restored by the loss of 53BP1 (Fig. 5). Moreover, we found that alovudine induces subnuclear 53BP1 foci more pronouncedly in *FEN1^−/−^*cells than in wild-type cells and these alovudine-induced 53BP1 foci significantly colocalize with the site of nascent DNA synthesis (Figs 6 and 7). These data indicate that Fen1 limits the toxic interaction of 53BP1 to alovudine-incorporated nascent Okazaki fragments.

We demonstrated that Fen1 counteracts 53BP1’s toxic action on Okazaki fragment maturation in the presence of alovudine. A possible mechanism underlying Fen1 mediated suppression of the toxic effect of 53BP1 is as follows: In the presence of Fen1, short flaps caused during Okazaki fragment maturation are efficiently digested by Fen1 endonuclease activity and incorporated alovudine in RNA primers should be efficiently eliminated (Supplementary Fig. S8A). In contrast, the loss of Fen1 results in the accumulation of the long flaps, leading to the binding of 53BP1 to displaced long RNA–DNA chimera in Okazaki fragment during lagging strand synthesis [16, 50, 51], where alovudine might be incorporated. This alovudine-incorporated Okazaki fragment might strongly trap 53BP1 thereby interfering removal of such long flaps by Dna2 or RNaseH1 thereby inducing DNA damage (Supplementary Fig. S8B). In the absence of both Fen1 and 53BP1, the accumulated long flaps due to the lack of Fen1 containing alovudine might be removed by the other removal system involved in Okazaki fragment maturation such as Dna2 or RNasse H1 [52–54] (Supplementary Fig. S8C). This seems indeed the case since we detected increased subnuclear 53BP1 foci formation in alovudine-treated *FEN1^−/−^* cells compared with untreated cell or alovudine-treated wild-type cells (Figs 6 and 7). Moreover, these 53BP1 foci significantly overlapped with γH2AX foci (Supplementary Fig. S6). We also demonstrated that Fen1 deficient cells show more 53BP1 foci which colocalize with the site of nascent DNA synthesis (EdU foci) (Fig. 7 and Supplementary Fig. S7). Recent study demonstrated that Fen1 similarly mitigates toxic effects of PARP-DNA complex induced by PARP inhibitor on the Okazaki fragment maturation [55]. This study adds the new mechanism of Fen1 in the suppression of toxic action of 53BP1 in the removal of alovudine-incorporated flaps in Okazaki fragments (Supplementary Fig. S8).

Fen1 is an essential tumor suppressor protein reported to be overexpressed in brain, testicular, and lung cancers [56]. The *FEN1-E160D* mutation in mice induces autoimmunity, chronic inflammation, and cancers, followed by the initiation and progression of cancer [57, 58]. The *FEN1-69GG* genotypes were also found to be significantly correlated with an increased risk of developing breast cancer, which defined *FEN1* as an important gene in human breast carcinogenesis [59]. In breast, ovarian, and gastric cancers, Fen1 has been attempted as a key biomarker [60, 61]. In our present study, we have described the crucial role of Fen1 in avoiding replication fork arrest as well as DNA damage upon alovudine treatment, thus implementing alovudine as a possible therapeutic drug for Fen1 deficient cancers.

## Supplementary Material

gkaf617_Supplemental_Files

## Data Availability

All data are in the manuscript and/or supporting information files. All raw data gained in this study are shown in S9_raw data.

## References

[1] Ogilvie KK, Slotin L, Rheault P Novel substrate of adenosine deaminase. Biochem Biophys Res Commun. 1971; 45:297–300. 10.1016/0006-291X(71)90817-5. 5168598

[2] Tsuda M, Terada K, Ooka M et al. The dominant role of proofreading exonuclease activity of replicative polymerase ϵ in cellular tolerance to cytarabine (Ara-C). Oncotarget. 2017; 8:33457–74. 10.18632/oncotarget.16508. 28380422 PMC5464882

[3] Mitsuya H, Yarchoan R, Broder S Molecular targets for AIDS therapy. Science. 1990; 249:1533–44. 10.1126/science.1699273. 1699273

[4] Schaeffer HJ, Beauchamp L, de Miranda P et al. 9-(2-hydroxyethoxymethyl) guanine activity against viruses of the herpes group. Nature. 1978; 272:583–5. 10.1038/272583a0. 205792

[5] Ghosn J, Quinson AM, Sabo N et al. Antiviral activity of low-dose alovudine in antiretroviral-experienced patients: results from a 4-week randomized, double-blind, placebo-controlled dose-ranging trial. HIV Med. 2007; 8:142–7. 10.1111/j.1468-1293.2007.00444.x. 17461857

[6] De Clercq E Emerging anti-HIV drugs. Expert Opin Emerging Drugs. 2005; 10:241–74. 10.1517/14728214.10.2.241. 15934866

[7] Hosen MB, Kawasumi R, Hirota K Dominant roles of BRCA1 in cellular tolerance to a chain-terminating nucleoside analog, alovudine. DNA Repair (Amst). 2024; 137:103668 10.1016/j.dnarep.2024.103668. 38460389

[8] Mirman Z, de Lange T 53BP1: a DSB escort. Genes Dev. 2020; 34:7–23. 10.1101/gad.333237.119. 31896689 PMC6938671

[9] Bunting SF, Callén E, Wong N et al. 53BP1 inhibits homologous recombination in Brca1-deficient cells by blocking resection of DNA breaks. Cell. 2010; 141:243–54. 10.1016/j.cell.2010.03.012. 20362325 PMC2857570

[10] Harrington JJ, Lieber MR DNA structural elements required for FEN-1 binding. J Biol Chem. 1995; 270:4503–8. 10.1074/jbc.270.9.4503. 7876218

[11] Murante RS, Henricksen LA, Bambara RA Junction ribonuclease: an activity in Okazaki fragment processing. Proc Natl Acad Sci USA. 1998; 95:2244–9. 10.1073/pnas.95.5.2244. 9482870 PMC19307

[12] Wu X, Li J, Li X et al. Processing of branched DNA intermediates by a complex of human FEN-1 and PCNA. Nucleic Acids Res. 1996; 24:2036–43. 10.1093/nar/24.11.2036. 8668533 PMC145902

[13] Liu Y, Kao HI, Bambara RA Flap endonuclease 1: a central component of DNA metabolism. Annu Rev Biochem. 2004; 73:589–615. 10.1146/annurev.biochem.73.012803.092453. 15189154

[14] Balakrishnan L, Gloor JW, Bambara RA Reconstitution of eukaryotic lagging strand DNA replication. Methods. 2010; 51:347–57. 20178844 10.1016/j.ymeth.2010.02.017 PMC2900510

[15] Stodola JL, Burgers PM Mechanism of lagging-strand DNA replication in eukaryotes. Adv Exp Med Biol. 2017; 1042:117–33. 29357056 10.1007/978-981-10-6955-0_6

[16] Leriche M, Bonnet C, Jana J et al. 53BP1 interacts with the RNA primer from Okazaki fragments to support their processing during unperturbed DNA replication. Cell Rep. 2023; 42:113412 10.1016/j.celrep.2023.113412. 37963016

[17] Helt CE, Wang W, Keng PC et al. Evidence that DNA damage detection machinery participates in DNA repair. Cell Cycle. 2005; 4:529–32. 10.4161/cc.4.4.1598. 15876866

[18] Liu Y, Wilson SH DNA base excision repair: a mechanism of trinucleotide repeat expansion. Trends Biochem Sci. 2012; 37:162–72. 10.1016/j.tibs.2011.12.002. 22285516 PMC3323758

[19] Sun H, He L, Wu H et al. The FEN1 L209P mutation interferes with long-patch base excision repair and induces cellular transformation. Oncogene. 2017; 36:194–207. 10.1038/onc.2016.188. 27270424 PMC5140775

[20] Yang F, Hu Z, Guo Z Small-molecule inhibitors targeting FEN1 for cancer therapy. Biomolecules. 2022; 12:1007 10.3390/biom12071007. 35883563 PMC9312813

[21] Zheng L, Dai H, Hegde ML et al. Fen1 mutations that specifically disrupt its interaction with PCNA cause aneuploidy-associated cancer. Cell Res. 2011; 21:1052–67. 10.1038/cr.2011.35. 21383776 PMC3129403

[22] Washif M, Ahmad T, Hosen MB et al. CTF18-RFC contributes to cellular tolerance against chain-terminating nucleoside analogs (CTNAs) in cooperation with proofreading exonuclease activity of DNA polymerase ϵ. DNA Repair (Amst). 2023; 127:103503 10.1016/j.dnarep.2023.103503. 37099849

[23] Hirota K, Ooka M, Shimizu N et al. XRCC1 counteracts poly(ADP ribose)polymerase (PARP) poisons, olaparib and talazoparib, and a clinical alkylating agent, temozolomide, by promoting the removal of trapped PARP1 from broken DNA. Genes Cells. 2022; 27:331–44. 10.1111/gtc.12929. 35194903 PMC9310723

[24] Buerstedde JM, Takeda S Increased ratio of targeted to random integration after transfection of chicken B cell lines. Cell. 1991; 67:179–88. 10.1016/0092-8674(91)90581-I. 1913816

[25] Yoshimura M, Kohzaki M, Nakamura J et al. Vertebrate POLQ and POLbeta cooperate in base excision repair of oxidative DNA damage. Mol Cell. 2006; 24:115–25. 10.1016/j.molcel.2006.07.032. 17018297 PMC1868411

[26] Ooka M, Abe T, Cho K et al. Chromatin remodeler ALC1 prevents replication-fork collapse by slowing fork progression. PLoS One. 2018; 13:e0192421 10.1371/journal.pone.0192421. 29408941 PMC5800655

[27] Martin RW, Orelli BJ, Yamazoe M et al. RAD51 up-regulation bypasses BRCA1 function and is a common feature of BRCA1-deficient breast tumors. Cancer Res. 2007; 67:9658–65. 10.1158/0008-5472.CAN-07-0290. 17942895

[28] Kikuchi K, Taniguchi Y, Hatanaka A et al. Fen-1 facilitates homologous recombination by removing divergent sequences at DNA break ends. Mol Cell Biol. 2005; 25:6948–55. 10.1128/MCB.25.16.6948-6955.2005. 16055708 PMC1190240

[29] Nakamura K, Sakai W, Kawamoto T et al. Genetic dissection of vertebrate 53BP1: a major role in non-homologous end joining of DNA double strand breaks. DNA Repair (Amst). 2006; 5:741–9. 10.1016/j.dnarep.2006.03.008. 16644291

[30] Rahman M, Hirota K, Kawasumi R Proofreading exonuclease activity of replicative polymerase epsilon promotes cellular tolerance to arabinosides in CTF18-dependent and -independent manner. Genome Instab Dis. 2024; 5:76–88. 10.1007/s42764-024-00124-w.

[31] Zhang LS, Honma M, Hayashi M et al. A comparative study of TK6 human lymphoblastoid and L5178Y mouse lymphoma cell lines in the in vitro micronucleus test. Mutation Research Letters. 1995; 347:105–15. 10.1016/0165-7992(95)00027-5. 7565900

[32] Rahman MR, Kawasumi R, Hirota K The flap endonuclease-1 mediated maturation of Okazaki fragments is critical for the cellular tolerance to remdesivir. DNA Repair (Amst). 2024; 144:103773 10.1016/j.dnarep.2024.103773. 39405747

[33] Kawasumi R, Abe T, Arakawa H et al. ESCO1/2’s roles in chromosome structure and interphase chromatin organization. Genes Dev. 2017; 31:2136–50. 10.1101/gad.306084.117. 29196537 PMC5749162

[34] Sasanuma H, Tsuda M, Morimoto S et al. BRCA1 ensures genome integrity by eliminating estrogen-induced pathological topoisomerase II-DNA complexes. Proc Natl Acad Sci USA. 2018; 115:E10642–51. 10.1073/pnas.1803177115. 30352856 PMC6233096

[35] Inomata Y, Abe T, Tsuda M et al. Division of labor of Y-family polymerases in translesion-DNA synthesis for distinct types of DNA damage. PLoS One. 2021; 16:e0252587 10.1371/journal.pone.0252587. 34061890 PMC8168857

[36] Fujita M, Sasanuma H, Yamamoto KN et al. Interference in DNA replication can cause mitotic chromosomal breakage unassociated with double-strand breaks. PLoS One. 2013; 8:e60043 10.1371/journal.pone.0060043. 23573231 PMC3616066

[37] Shimizu N, Ooka M, Takagi T et al. Distinct DNA damage spectra induced by ionizing radiation in normoxic and hypoxic cells. Radiat Res. 2015; 184:442–8. 10.1667/RR14117.1. 26430822

[38] Braafladt S, Reipa V, Atha DH The Comet Assay: automated imaging methods for improved analysis and reproducibility. Sci Rep. 2016; 6:32162. 27581626 10.1038/srep32162 PMC5007470

[39] Barbé L, Lam S, Holub A et al. AutoComet: a fully automated algorithm to quickly and accurately analyze comet assays. Redox Biol. 2023; 62:102680 10.1016/j.redox.2023.102680. 37001328 PMC10090439

[40] El-Zaria ME, Nakamura H New strategy for synthesis of mercaptoundecahydrododecaborate derivatives via click chemistry: possible boron carriers and visualization in cells for neutron capture therapy. Inorg Chem. 2009; 48:11896–902. 10.1021/ic902033c. 19928788

[41] Fernandes SA, Angelidaki DD, Nüchel J et al. Spatial and functional separation of mTORC1 signalling in response to different amino acid sources. Nat Cell Biol. 2024; 26:1918–33. 10.1038/s41556-024-01523-7. 39385049 PMC11567901

[42] Schindelin J, Arganda-Carreras I, Frise E et al. Fiji: an open-source platform for biological-image analysis. Nat Methods. 2012; 9:676–82. 10.1038/nmeth.2019. 22743772 PMC3855844

[43] Costes SV, Daelemans D, Cho EH et al. Automatic and quantitative measurement of protein-protein colocalization in live cells. Biophys J. 2004; 86:3993–4003. 10.1529/biophysj.103.038422. 15189895 PMC1304300

[44] Murai J, Pommier Y BRCAness, homologous recombination deficiencies, and synthetic lethality. Cancer Res. 2023; 83:1173–4. 10.1158/0008-5472.CAN-23-0628. 37057596

[45] Sonoda E, Sasaki MS, Morrison C et al. Sister chromatid exchanges are mediated by homologous recombination in vertebrate cells. Mol Cell Biol. 1999; 19:5166–9. 10.1128/MCB.19.7.5166. 10373565 PMC84359

[46] Noordermeer SM, Adam S, Setiaputra D et al. The shieldin complex mediates 53BP1-dependent DNA repair. Nature. 2018; 560:117–21. 10.1038/s41586-018-0340-7. 30022168 PMC6141009

[47] Quinet A, Carvajal-Maldonado D, Lemacon D et al. DNA fiber analysis: mind the gap!. Methods Enzymol. 591:55–82. 28645379 10.1016/bs.mie.2017.03.019

[48] Sun H, Ma L, Tsai YF et al. Okazaki fragment maturation: DNA flap dynamics for cell proliferation and survival. Trends Cell Biol. 2023; 33:221–34. 10.1016/j.tcb.2022.06.014. 35879148 PMC9867784

[49] Mórocz M, Gali H, Raskó I et al. Single cell analysis of human RAD18-dependent DNA post-replication repair by alkaline bromodeoxyuridine comet assay. PLoS One. 2013; 8:e70391 10.1371/journal.pone.0070391. 23936422 PMC3735594

[50] Liu B, Hu J, Wang J et al. Direct visualization of RNA–DNA primer removal from Okazaki fragments provides support for flap cleavage and exonucleolytic pathways in eukaryotic cells. J Biol Chem. 2017; 292:4777–88. 10.1074/jbc.M116.758599. 28159842 PMC5377794

[51] Shi G, Yang C, Wu J et al. DNA polymerase δ subunit Pol32 binds histone H3–H4 and couples nucleosome assembly with Okazaki fragment processing. Sci Adv. 2024; 10:eado1739. 39121223 10.1126/sciadv.ado1739 PMC11313866

[52] Kang YH, Lee CH, Seo YS Dna2 on the road to Okazaki fragment processing and genome stability in eukaryotes. Crit Rev Biochem Mol Biol. 2010; 45:71–96. 10.3109/10409230903578593. 20131965

[53] Kao HI, Bambara RA The protein components and mechanism of eukaryotic Okazaki fragment maturation. Crit Rev Biochem Mol Biol. 2003; 38:433–52. 10.1080/10409230390259382. 14693726

[54] Zheng L, Meng Y, Campbell JL et al. Multiple roles of DNA2 nuclease/helicase in DNA metabolism, genome stability and human diseases. Nucleic Acids Res. 2020; 48:16–35. 10.1093/nar/gkz1101. 31754720 PMC6943134

[55] Vaitsiankova A, Burdova K, Sobol M et al. PARP inhibition impedes the maturation of nascent DNA strands during DNA replication. Nat Struct Mol Biol. 2022; 29:329–38. 10.1038/s41594-022-00747-1. 35332322 PMC9010290

[56] Nikolova T, Christmann M, Kaina B FEN1 is overexpressed in testis, lung and brain tumors. Anticancer Res. 2009; 29:2453–9. 19596913

[57] Zheng L, Dai H, Qiu J et al. Disruption of the FEN-1/PCNA interaction results in DNA replication defects, pulmonary hypoplasia, pancytopenia, and newborn lethality in mice. Mol Cell Biol. 2007; 27:3176–86. 10.1128/MCB.01652-06. 17283043 PMC1899923

[58] Zheng L, Dai H, Zhou M et al. Fen1 mutations result in autoimmunity, chronic inflammation and cancers. Nat Med. 2007; 13:812–9. 10.1038/nm1599. 17589521

[59] Lv Z, Liu W, Li D et al. Association of functional FEN1 genetic variants and haplotypes and breast cancer risk. Gene. 2014; 538:42–5. 10.1016/j.gene.2014.01.025. 24440783

[60] Abdel-Fatah TM, Russell R, Albarakati N. et al. Genomic and protein expression analysis reveals flap endonuclease 1 (FEN1) as a key biomarker in breast and ovarian cancer. Molecular Oncology. 2014; 8:1326–38. 10.1016/j.molonc.2014.04.009. 24880630 PMC4690463

[61] Wang K, Xie C, Chen D Flap endonuclease 1 is a promising candidate biomarker in gastric cancer and is involved in cell proliferation and apoptosis. Int J Mol Med. 2014; 33:1268–74. 10.3892/ijmm.2014.1682. 24590400

