# Supplementary material for: The flap endonuclease-1 promotes cellular tolerance to a chain-terminating nucleoside analog, alovudine, by counteracting the toxic effect of 53BP1
Source: Nucleic Acids Res. 2025 Jul 18;53(13):gkaf617. doi: 10.1093/nar/gkaf617 (PMC12270548; doi:10.1093/nar/gkaf617)
Supplement: gkaf617_Supplemental_Files [file gkaf617_supplemental_files.zip › Supplementary information.pdf]

Supplementary information

The flap endonuclease-1 promotes cellular tolerance to a chain-terminating nucleoside analog, alovudine by counteracting the toxic effect of 53BP1

Md Bayejid Hosen, Ryotaro Kawasum, and Kouji Hirota

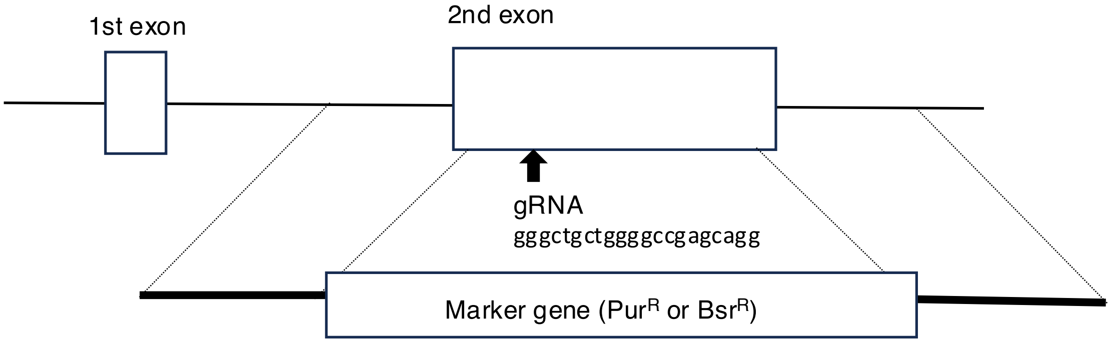

Fig. S1 Generation of *FEN1*$^{-/-}$ TK6 cells

FEN1 gene disruption in human TK6 cells. Knockout constructs are shown below the locus. The boxes represent exons. The thick lines show the genomic region amplified for targeting vector arms. The indicated gRNA sequence was inserted into the BbsI site of pX330 (Cat# 42230, Addgene, US). pX330 expresses gRNA under the control of the U6 promoter, and Cas9 under the control of the chicken β-actin promoter. pX330-gRNA and the two indicated targeting vectors were transfected into TK6 cells as described in materials and methods. At 24 h after the transfection, appropriate selection reagents were added to select cells carrying maker genes.

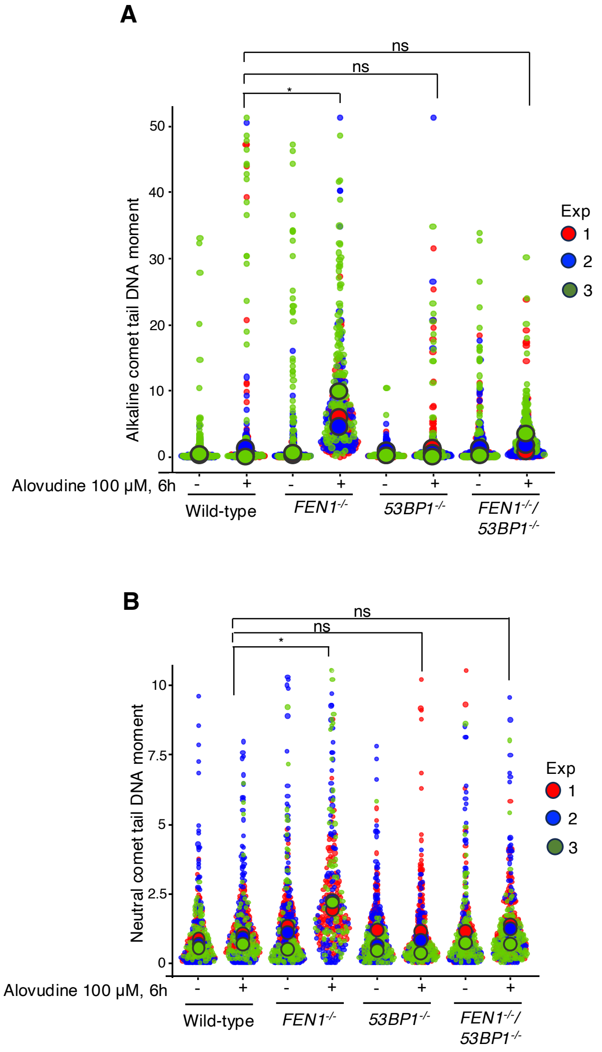

Fig. S2 Fen1 suppresses 53BP1 and reduced alovudine induced DNA breaks

(A−B) DNA breaks in genomic DNA quantified by alkaline comet assay (A) and neutral comet assays (B) in DT40 cells with the indicated genotypes following incubation (6 h) with alovudine (100 μM). The scatter plots represent the comet tail moments per cell. The plots were generated using SuperPlotsOfData (https://huygens.science.uva.nl/SuperPlotsOfData/). For each sample, scatter plots are the tail moments of more than 300 individual cells combined from three independent experiments and the circles represent the medians. The p-value was calculated using Student's t-test. ns, not significant; $p > 0.05$.

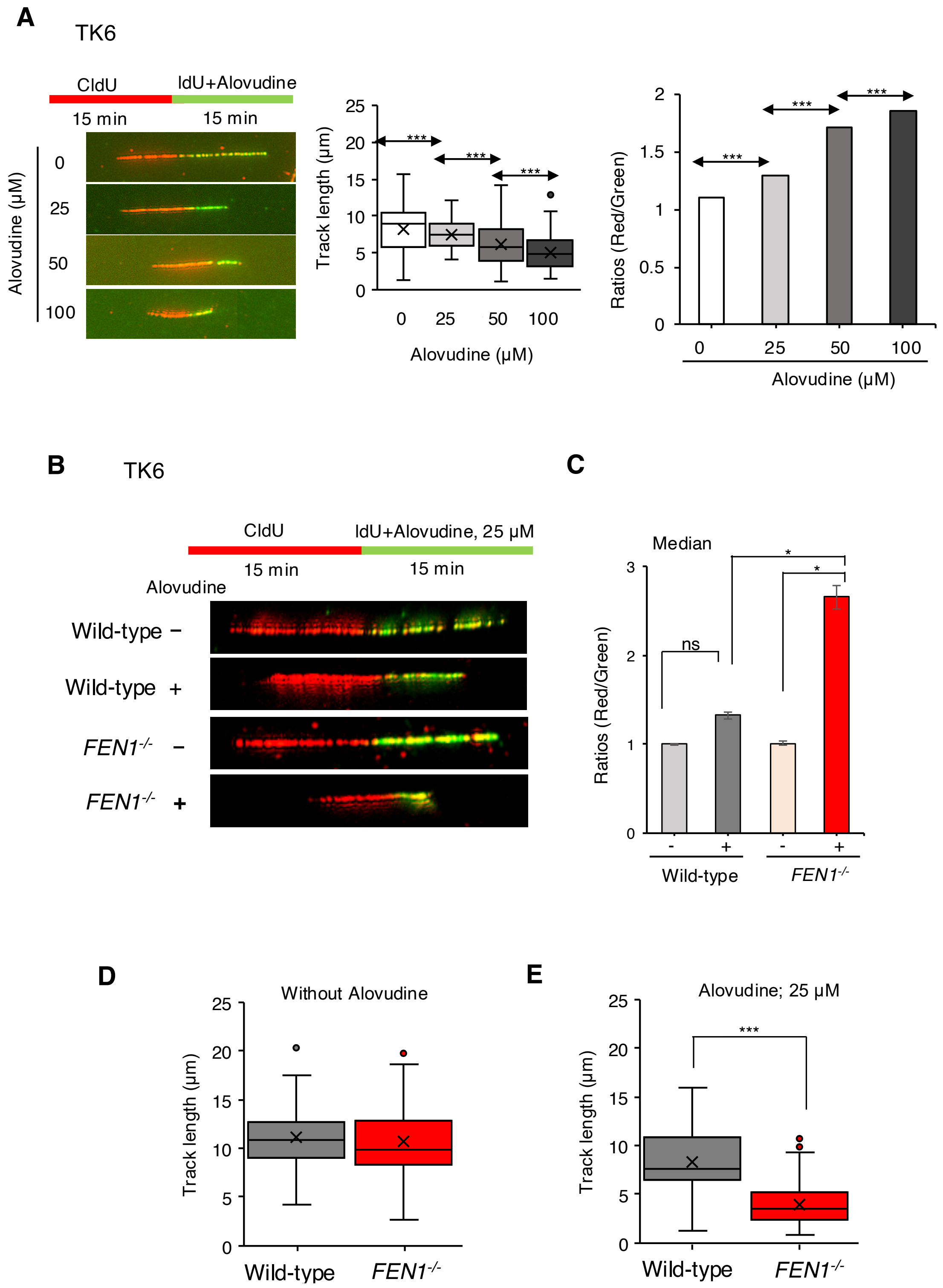

Fig. S3 Fen1 maintains replication fork speed upon alovudine in TK6 cells

(A) Determination of alovudine doses that exhibit activity to linearly reduce the rate of replication fork progression in TK6 cells. Indicated cells were labeled sequentially with CldU and IdU for 15

min each time and treated with alovudine during IdU labeling. Lengths of the CldU and IdU tracks were measured, and the CldU/IdU ratio for each replication fork was calculated for at least 50 forks. Representative images showing DNA fibers are presented. The quantification results of fork length are shown. (Middle line) Median, (box) 25th and 75th percentiles, and (bars) 10th and 90th percentiles. The medians of the quantified CldU/IdU ratio were obtained from three independent analyses and means and SDs of ratios are presented. All statistical analyses were performed by two two-tailed Student's *t*-test. ns: not significant. (B) Schematic representation of the experimental protocol to monitor fork speed. TK6 cells were sequentially pulse-labeled with CldU and IdU for 15 min each. Cells were exposed to 25 μM alovudine in the second labeling. Representative images showing DNA fibers from TK6 cells. (C) Lengths of the CldU and IdU tracks were measured, and the CldU/IdU ratio for each replication fork was calculated for at least 50 forks. The medians of CldU/IdU ratio were obtained from two independent analyses and means and SDs of ratios were presented. All statistical analyses were performed by two two-tailed Student's *t*-test. ns: not significant. $*p < 0.05$. (C-D) Quantification results of fork speed in unperturbed condition (D) and alovudine-challenged condition (E). The black line indicates the median. All statistical analyses were performed using the Mann-Whitney-Wilcoxon test. ns, not significant; $*p < 0.05$, $**p < 0.01$, $***p < 0.001$.

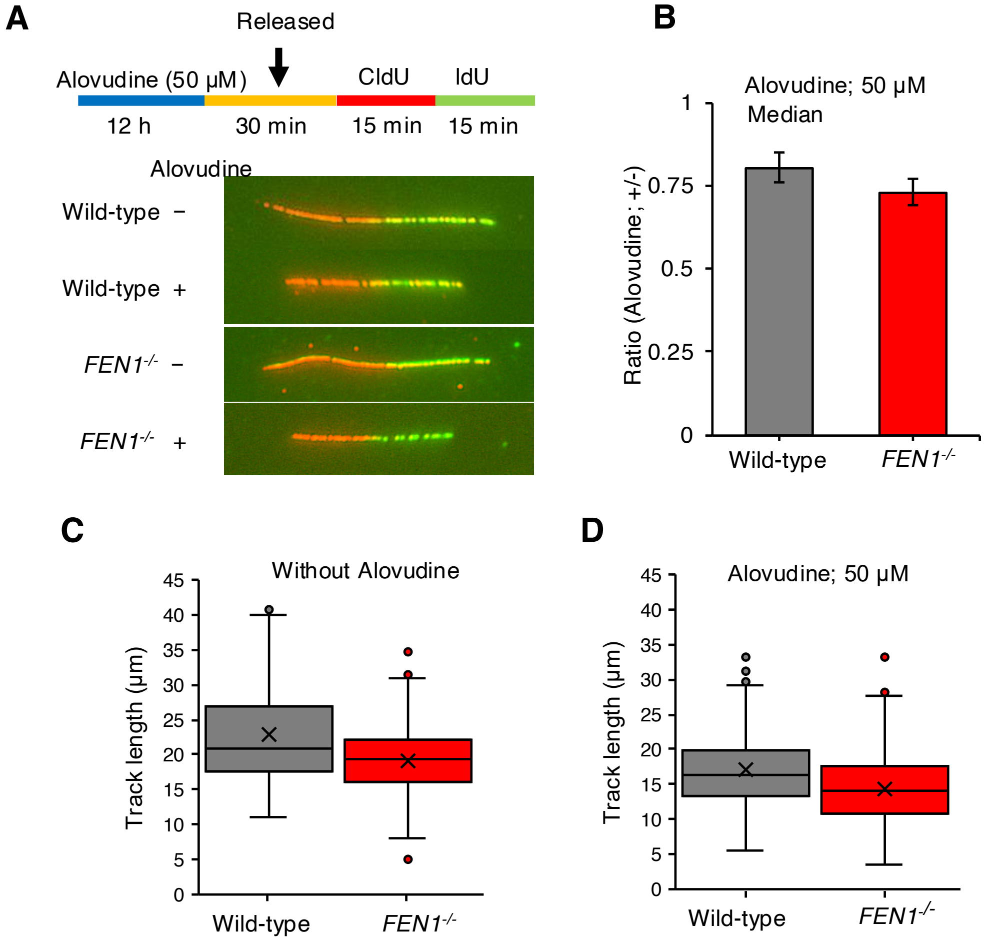

Fig. S4 The not important role of Fen1 in the maintenance of replication fork in the alovudine-incorporated template

Schematic representation of the experimental protocol to monitor fork speed. DT40 were cultured in a medium containing 50 µM alovudine for 12 h, followed by release in a drug-free medium and culturing for 30 min. The cells were sequentially pulse-labeled with CldU and IdU for 15 min each. Representative images showing DNA fibers from DT40 cells. (B)The ratio between unperturbed replication speed and alovudine-challenged condition was calculated for at least 50 forks. The medians of the ratio were obtained from three independent analyses and means and SDs of ratios were presented. All statistical analyses were performed by two two-tailed Student's *t*-test. ns: not significant (C-D) Quantification results of fork length in DT40 cells in unperturbed condition (C) and alovudine-challenged condition (D).

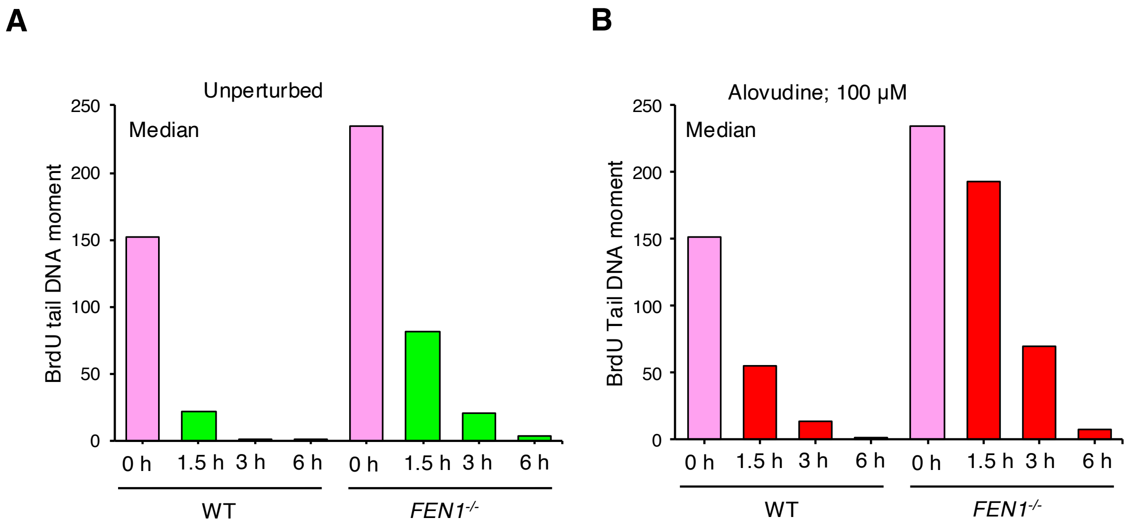

Fig. S5 Important role of Fen1 on the maturation of Okazaki fragment

(A−B) BrdU comet tail moments were quantified in the indicated cells following incubation with alovudine (100 μM). BrdU comet tail moments were scored by staining with anti-BrdU antibodies following a 0.5 h BrdU pulse label and subsequent 1.5 h, 3 h, and 6 h chasing. For each sample, bar plots are the tail moments of more than 50 individual nuclei and the bars represent the medians.

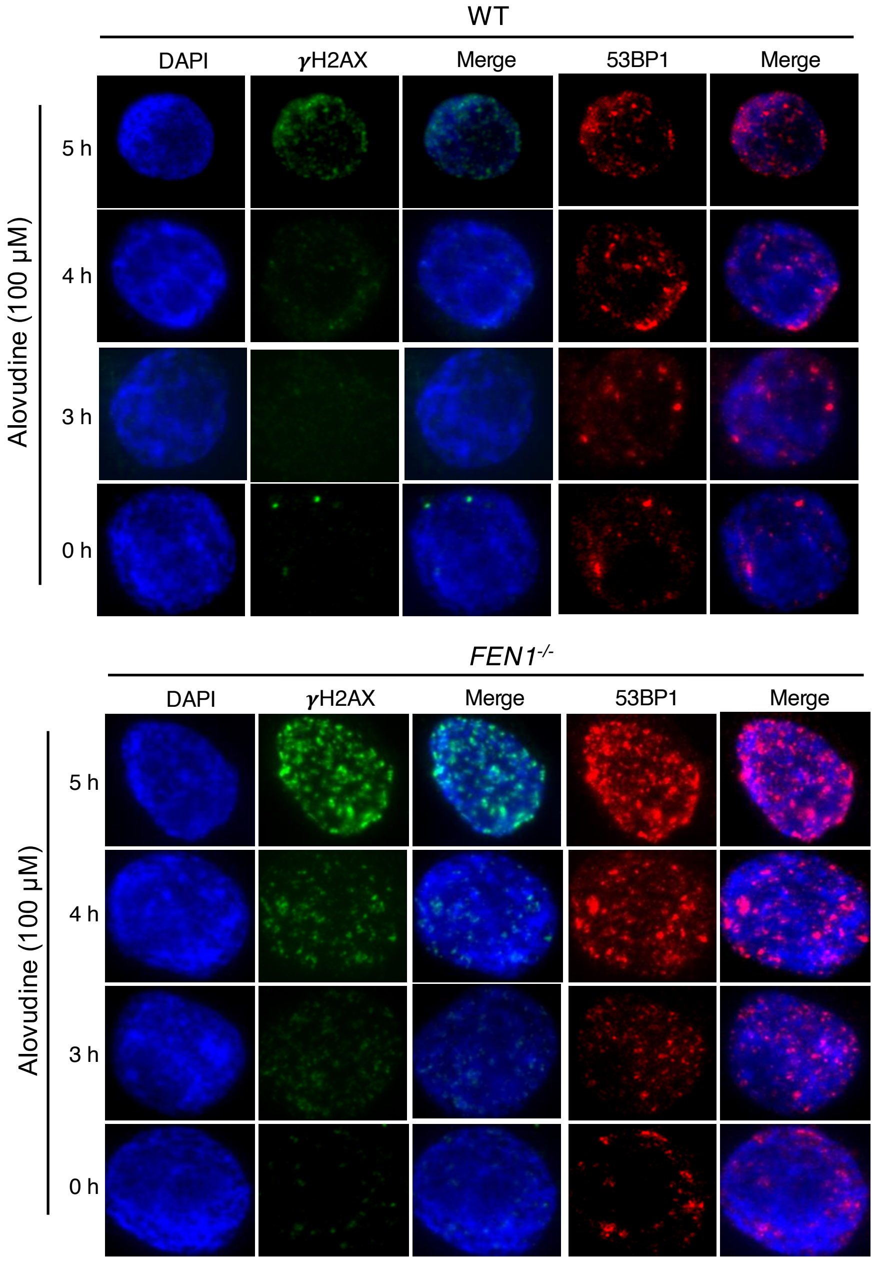

Fig. S6 Representative fluorescence microscopic images of 53BP1 and γH2AX foci

Representative fluorescence microscopic images of 53BP1 and γH2AX foci in the indicated cell lines at different periods of alovudine exposure.

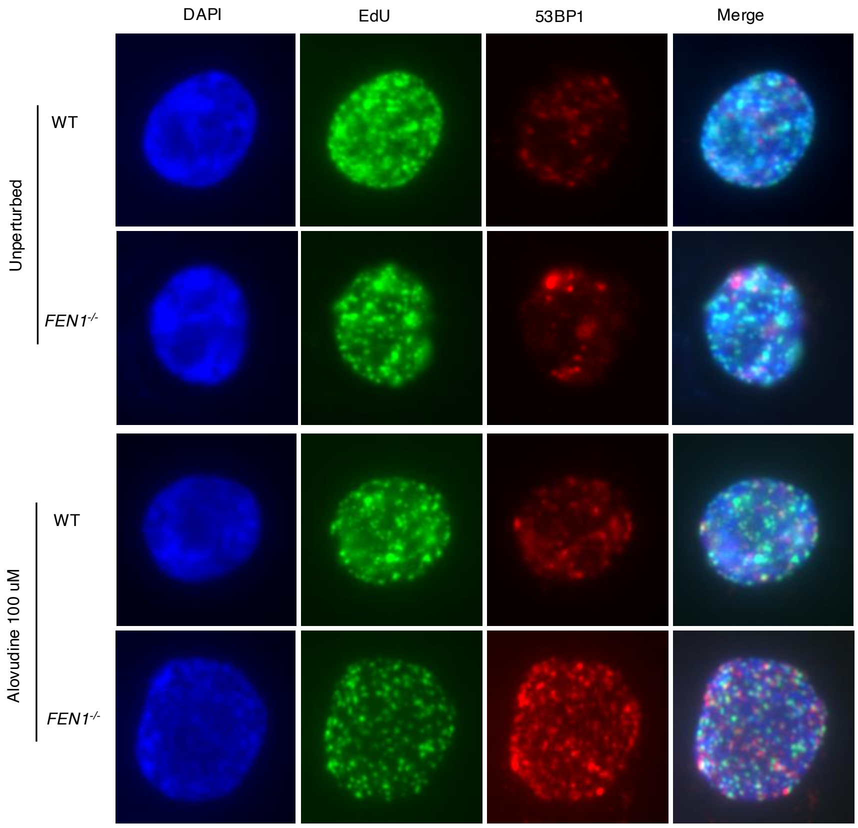

Fig. S7 Representative fluorescence microscopic images of 53BP1 and EdU foci

Representative fluorescence microscopic images of 53BP1 and EdU foci in the indicated cell lines were shown. Indicated cells were treated as in Fig. 7A.

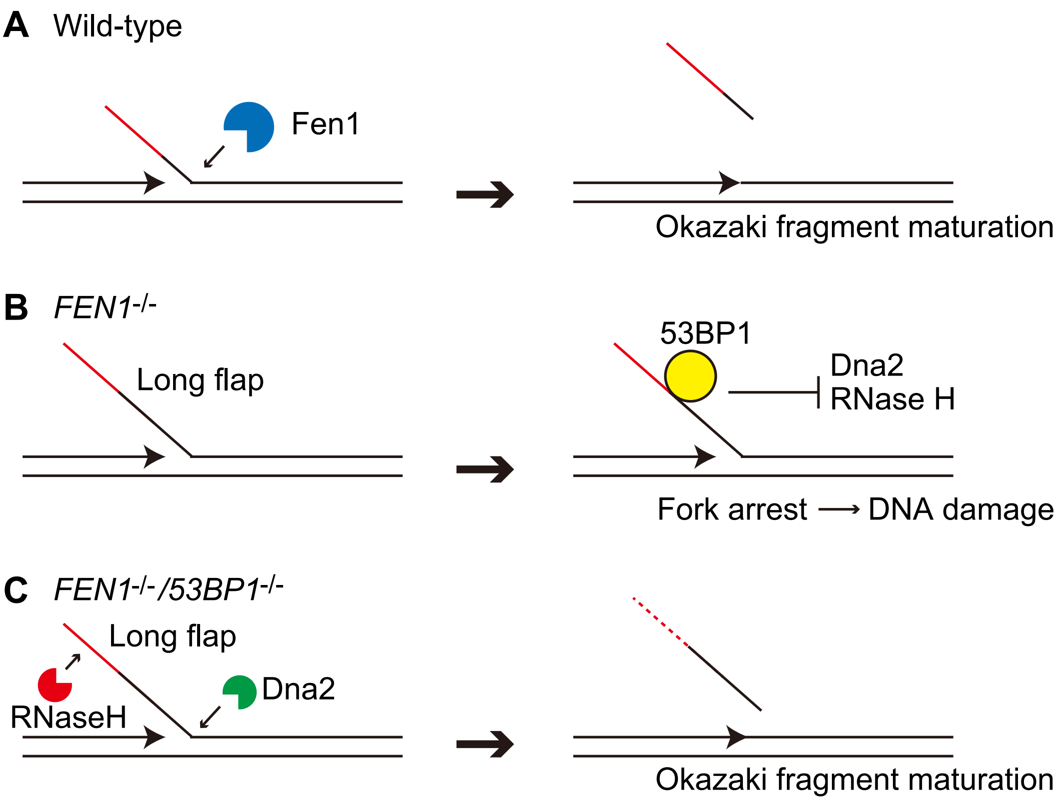

Fig. S8 A model

(A) In wild-type cells, Fen1 removes alovudine-incorporated nascent 3'-flapped DNA and promotes Okazaki fragment maturation. (B) In Fen1 deficient cells, alovudine-incorporated nascent 3'-flapped DNA was not removed in a timely manner, and the resultant long flap containing alovudine traps 53BP1, thereby suppressing alternative Okazaki fragment maturation systems such as Dna2 and RNaseH, leading to DNA damage. (C) In the absence of Fen1 and 53BP1, alternative Okazaki fragment maturation systems such as Dna2 and RNaseH execute Okazaki fragment maturation via degradation of RNA primer (right, dashed line) and removal of 3' flapped nascent DNA.
